# Molecular Characterization of Human Pathogenic Bunyaviruses of the Nyando and Bwamba/Pongola Virus Groups Leads to the Genetic Identification of Mojuí dos Campos and Kaeng Khoi Virus

**DOI:** 10.1371/journal.pntd.0003147

**Published:** 2014-09-04

**Authors:** Allison Groseth, Veena Mampilli, Carla Weisend, Eric Dahlstrom, Stephen F. Porcella, Brandy J. Russell, Robert B. Tesh, Hideki Ebihara

**Affiliations:** 1 Molecular Virology and Host-Pathogen Interaction Unit, Laboratory of Virology, Division of Intramural Research, National Institute of Allergy and Infectious Diseases, National Institutes of Health, Hamilton, Montana, United States of America; 2 RML Genomics Unit, Research Technology Branch, National Institute of Allergy and Infectious Diseases, National Institutes of Health, Hamilton, Montana, United States of America; 3 Division of Vector-Borne Infectious Diseases, Centers for Disease Control & Prevention, Fort Collins, Colorado, United States of America; 4 Department of Pathology and Center for Biodefense and Emerging Infectious Diseases, University of Texas Medical Branch, Galveston, Texas, United States of America; Aix Marseille University, Institute of Research for Development, and EHESP School of Public Health, France

## Abstract

**Background:**

Human infection with Bwamba virus (BWAV) and the closely related Pongola virus (PGAV), as well as Nyando virus (NDV), are important causes of febrile illness in Africa. However, despite seroprevalence studies that indicate high rates of infection in many countries, these viruses remain relatively unknown and unstudied. In addition, a number of unclassified bunyaviruses have been isolated over the years often with uncertain relationships to human disease.

**Methodology/Principal Findings:**

In order to better understand the genetic and evolutionary relationships among orthobunyaviruses associated with human disease, we have sequenced the complete genomes for all 3 segments of multiple strains of BWAV (n = 2), PGAV (n = 2) and NDV (n = 4), as well as the previously unclassified Mojuí dos Campos (MDCV) and Kaeng Khoi viruses (KKV). Based on phylogenetic analysis, we show that these viruses populate 2 distinct branches, one made up of BWAV and PGAV and the other composed of NDV, MDCV and KKV. Interestingly, the NDV strains analyzed form two distinct clades which differed by >10% on the amino acid level across all protein products. In addition, the assignment of two bat-associated bunyaviruses into the NDV group, which is clearly associated with mosquito-borne infection, led us to analyze the ability of these different viruses to grow in bat (RE05 and Tb 1 Lu) and mosquito (C6/36) cell lines, and indeed all the viruses tested were capable of efficient growth in these cell types.

**Conclusions/Significance:**

On the basis of our analyses, it is proposed to reclassify the NDV strains ERET147 and YM176-66 as a new virus species. Further, our analysis definitively identifies the previously unclassified bunyaviruses MDCV and KKV as distinct species within the NDV group and suggests that these viruses may have a broader host range than is currently appreciated.

## Author Summary

Bunyavirus infections cause febrile illnesses of varying severity worldwide; however, despite their public health importance most remain relatively unstudied. In order to clarify the genetic relationships among African orthobunyaviruses associated with human infection, we have sequenced multiple strains of Bwamba (BWAV), Pongola (PGAV) and Nyando virus (NDV). Based on genetic analysis we showed that, while different BWAV and PGAV virus strains are closely related, NDV strains were highly variable and warrant classification as two distinct virus species. In addition, sequencing of the previously unclassified Mojuí dos Campos (MDCV) and Kaeng Khoi (KKV) viruses showed that both are closely related to NDV. This was unexpected considering that these viruses were isolated in South America and Southeast Asia, respectively, and are mainly associated with bats. Further, our experiments also indicated that BWAV and PGAV, as well as NDV, MDCV and KKV, are able to infect both bat and mosquito cell lines, suggesting that ecological studies focusing on these potential host and vector species are warranted. In the future, the availability of complete genetic information for these viruses, together with an understanding of their genetic relationships, will aid in better defining the distribution and public health impact of these viruses.

## Introduction

The *Bunyaviridae* are a large, diverse group of more than 350 viruses divided into 5 genera, of which more than 150 belong to the genus *Orthobunyavirus* [1]. Importantly, these viruses represent a significant cause of arthropod-borne human disease worldwide, with infection often associated with a febrile and/or encephalitic illness, and in rare cases also hemorrhagic manifestations [2]. However, despite their importance for public health, from both a genetic and an evolutionary standpoint the family has been only poorly characterized.

**Figure 1:**
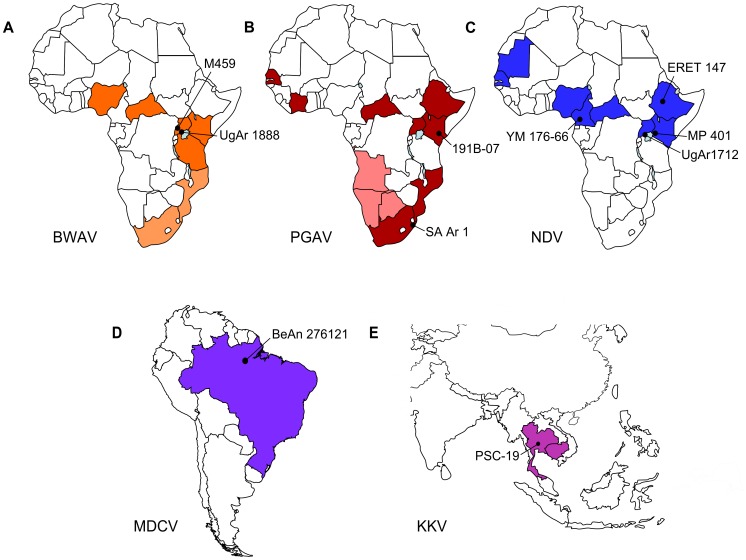
Geographic distribution of viruses used in this study. Distribution of (A) Bwamba virus, (B) Pongola virus, (C) Nyando virus, (D) Mojuí dos Campos virus, and (E) Kaeng Khoi virus. Countries in which these viruses have been isolated (dark colors) and/or where specific antibodies to these viruses have been detected (light colors) are shown in orange (BWAV), red (PGAV), blue (NDV), purple (MDCV), and pink (KKV), respectively. The geographic location from which the virus strains used in the present study were isolated, are indicated with black dots. The strain names for these isolates are also indicated.

In addition to the well-known agents of human bunyavirus disease in Africa, such as Rift Valley Fever virus (RVFV; genus *Phlebovirus*) and Crimean-Congo Hemorrhagic fever virus (CCHFV; genus *Nairovirus*), there are a number of viruses in the genus *Orthobunyavirus* that are also agents of human disease, with many of them being highly prevalent within their endemic areas. Among these viruses, by far the most prevalent appears to be Bwamba virus (BWAV), which has been reported to be among the most common arthropod-borne diseases in Africa [3]. Infection is associated with a relatively non-specific febrile illness that, while usually self-limiting, is frequently associated with exanthema and can include meningeal involvement [4]. However, recently a group of 14 fatal cases of BWAV infection with hemorrhagic complications, particularly bleeding from the oral mucosa and into the gastrointestinal tract, were reported during an outbreak among Rwandan refugees [4], indicating that BWAV infection can also be associated with very severe disease manifestations. To date a total of only 21 human cases of BWAV infection have been reported from various countries (including Uganda [4], [5], Central African Republic [6], Kenya [7] and Tanzania [4]). However, virus isolation and/or serological studies suggest that this virus circulates in several additional countries (i.e. Mozambique, South Africa and Nigeria) (Figure 1A) and show that seropositivity exceeds 90% in some populations [8]–[11]. Thus, these data clearly suggest that a lack of concerted surveillance efforts, together with the frequency of infections with other pathogens causing febrile illness in the affected areas, have contributed to under-reporting, and as a result an under-appreciation of the disease burden imposed by BWAV infection.

Interestingly, a closely related virus, Pongola virus (PGAV), has been isolated from mosquitoes (*Aedes circumluteolus*) in South Africa [9] and appears to have been responsible for a single reported case of human infection associated with febrile illness in Uganda [12]. While serological studies also indicate a high prevalence (9–26%) of PGAV infection in several countries (i.e. South Africa, Mozambique, Namibia, Botswana, Angola [8], [13]–[15]), it must be noted this virus is highly cross-reactive with BWAV in many serological tests [5], [9], [16], and that it has historically been difficult to distinguish the distributions and relative abundance of these two viruses; particularly since the affected geographical regions appear to substantially overlap (Figure 1A and B). As a consequence of this high degree of serological cross-reactivity, these viruses are classified together into a single serogroup.

Similar to BWAV and PGAV, Nyando virus (NDV) is also capable of causing moderate-severe febrile disease in humans. Although to date only a single human case with multiphasic fever, myalgia and vomiting has been reported from the Central African Republic [6], [17], again serological studies indicate a high level of seroprevalence in many countries, including Kenya, Uganda and Senegal [18], [19] (Figure 1C). Together with repeated isolations from mosquito pools [17], [18], [20] these findings indicate that, as with many of the African orthobunyaviruses, NDV virus might also be much more prevalent than is currently appreciated. Intriguingly, the limited sequencing data previously available for this virus suggests that Nyando virus (strain ArB16055; GenBank Accession AM709781) is closely related to Bunyamwera virus [21], a finding that appears to be at odds with the lack of serological cross-reactivity between these viruses. Indeed Nyando virus is classified into a distinct serogroup (Nyando serogroup), of which it is presently the only member [22].

Until now, bunyavirus classification has relied almost exclusively on serological testing, which may include complement fixation, hemagglutination inhibition, immunofluorescence assay and/or viral neutralization assays, and on the basis of these assays, the genus *Orthobunyavirus* is presently divided into 18 serogroups [22]. However, cross-reactivity between viruses is common and often limits our ability to make definitive identifications based on these methods [23], [24]. Further, many other bunyaviruses remain uncharacterized as a result of an inability to assign them a position within this serological classification [25]. Examples of such unclassified bunyaviruses include Mojuí dos Campos virus (MDCV), which was isolated from an unknown bat species in Brazil [26] (Figure 1D), and Kaeng Khoi virus (KKV), which has been isolated from bats (*Tadarida plicata* and *Taphozous theobaldi*) in Thailand and Cambodia [27]–[29] as well as from bedbugs (*Stricticimex parvus* and *Cimex insuetus*) [27] (Figure 1E). While no informative serological relationships could be established for KKV [29], MDCV was originally observed to show some cross-reactivity with Nyando virus as well as San Angelo virus (California encephalitis serogroup) [25], raising the possibility that it may be related to one or both of these viruses.

While genome sequencing is known to be both a rapid and accurate means of identifying viruses, and is indeed the standard for the identification of most other virus families, it is dependent on the availability of sufficient pre-existing sequence data, something that is presently lacking for bunyaviruses. Indeed, where genetic analysis of these viruses has been performed it is often extremely limited and focuses almost exclusively on the S-segment. However, this issue has been increasingly recognized within the field and is beginning to be addressed, particularly as a result of large-scale *de novo* sequencing efforts [30]–[32]. In light of the tri-segmented genome structure of these viruses, which allows them to evolve by both antigenic drift and antigenic shift (i.e. reassortment), this leaves us with an incomplete understanding of the exact identity of many bunyaviruses, and the relationships and diversity that exists among them. However, without substantial improvements in the availability of genetic information for these viruses, such determinations will be difficult, if not impossible.

In order to improve our understanding of the evolutionary relationships and genetic diversity among orthobunyaviruses causing human disease in Africa, and particularly the viruses of the Bwamba/Pongola virus and Nyando virus serogroups, we have undertaken the complete genome sequencing of multiple strains of each of these viruses. In addition, we have determined the first complete sequences of MDCV and KKV, which has allowed their definitive identification, and based on the genetic relationships identified in our analyses, we have begun to explore the possibility that additional host and vector species may be involved in the ecology of these viruses.

## Materials and Methods

### Cells and Viruses

Vero E6 (African green monkey kidney, ATCC CRL-1586), Tb 1 Lu (*Tadarida brasiliensis* lung, ATCC CCL-88) and RE05 (*Rousettus aegyptiacus* fetus; kindly provided by Ingo Jordan, ProBioGen AG [33]) cells were grown in Dulbecco's modified Eagle's medium (DMEM; Sigma-Aldrich), supplemented with 10% fetal calf serum (FCS; Life Technologies), 2 mM L-glutamine, 50 U/ml penicillin and 50 µg/ml streptomycin (Life Technologies) at 37°C in the presence of 5% CO_2_. C6/36 (*Aedes albopictus* larva, ATCC CRL-1660) were grown in Eagle's Minimum Essential Medium (EMEM; Life Technologies) supplemented with 10% fetal calf serum (FCS; Life Technologies), 2 mM L-glutamine, 50 U/ml penicillin and 50 µg/ml streptomycin (Life Technologies) at 28°C in the presence of 5% CO_2_.

The virus strains used in this study were kindly provided by the Centers for Disease Control and Prevention (CDC), Division of Vector-Borne Diseases (DVBD) arbovirus reference collection and the World Reference Center for Emerging Viruses and Arboviruses (WRCEVA) arthropod-borne virus reference collection. Information regarding their origin is summarized in Table 1.

**Table 1:** Virus strains used in this study.

Virus	Strain	Isolate Information			Ref.
		Source	Location	Date	
Bwamba virus (BWAV)	M459	Human	Bwamba County, Uganda	1937	[5]
	UgAr1888	*Anopheles funestus*	Rakai District, Uganda	1996	[51]
Pongola virus (PGAV)	SA Ar 1	*Aedes circumluteolus*	Natal, South Africa	1955	[9]
	191B-07	*Aedes mcintosh/circumluteolus*	Garissa, Kenya	2006	[45]
Nyando virus (NDV)	MP401	*Anopheles funestus*	Ramulla, Kenya	1959	[18]
	UgAr1712	*Anopheles funestus*	Rakai District, Uganda	1996	[51]
	ERET147	*Eretmapodites spp.*	Manéra, Ethiopia	1963	[52]
	YM176-66	*Aedes spp.*	Ofoumselek, Cameroon	1966	[48]
Mojuí dos Campos virus (MDCV)	BeAn 276121	Bat (undetermined species)	Pará, Brazil	1976	[26]
Kaeng Khoi virus (KKV)	PSC-19	Bat(*Tadarida plicata*)	Kaeng Khoi District, Thailand	1969	[27]

Virus stocks were grown in Vero E6 cells in DMEM supplemented with 2% FCS, 2 mM L-glutamine, 50 U/ml penicillin and 50 µg/ml streptomycin and 10 µg/ml Mycokill AB (GE Healthcare). Virus growth was monitored based on the appearance and progression of cytopathic effect (CPE) in cells. When advanced CPE was observed (50–75% of cells detached), the culture supernatants were harvested for RNA isolation.

### RNA Isolation

Cell culture supernatants from infected cells were spun twice at 1,000× *g* for 5 min at 4°C to pellet cell debris. For Sanger sequencing, RNA was then extracted using the QIAamp viral RNA extraction kit (Qiagen) according to the manufacturer's directions. Alternatively, for Next Generation sequencing (NGS), samples were further purified and concentrated through a centrifugal filtration device (Millipore) prior to RNA extraction, as previously described [34].

### Next Generation Sequencing and Data Analysis

cDNA for NGS was synthesized using a previously described modification [34] of the protocol described by Palacios *et al.* [35]. Briefly, first strand cDNA was synthesized using the Superscript III Reverse Transcriptase system (Life Technologies) with 100–1,000 ng of total RNA using a random octamer linked to a defined 17-mer primer (5′-GTT TCC CAG TAG GTC TCN NNN NNN N-3′). RNA was then hydrolyzed in NaOH and the single-stranded cDNA (ss-cDNA) products purified using the QIAquick system (Qiagen). The resulting ss-cDNAs were randomly amplified using a 1∶9 mixture of the arbitrary 17-octamer primer and a primer targeting a specific 17-mer sequence (5′- CGC CGT TTC CCA GTA GGT CTC-3′). The resulting ss-cDNA templates were used as template for PCR using Platinum Taq polymerase. PCR products were purified using the QIAquick kit following the manufacturer's protocol (Qiagen) and used as template for sequencing on the 454 Titanium FLX sequencer (454/Roche Life Sciences).

cDNA samples were quantitated using Picogreen reagent (Life Technologies) and prepared according to the Rapid Library Preparation Method Manual – GS FLX Titanium Series October 2009 (454 Life Sciences). A multiplex was titrated in medium volume emulsion (MVE) format to determine the optimal copy-per-bead ratio (CPB) which produced the best sequencing quality. A 454 Titanium sequencing run was then performed using 1 CPB.

Genomic viral sequences were produced on the 454 FLX sequencer and *de novo* assembled using GS De Novo Assembler v2.6 (454 Life Sciences) and CLC Genomics Workbench 4.0 (CLC Bio). Translated BLAST (blastx) was performed to remove non-viral contaminants and the initial assembly was performed using Sequencher v5.0 (Gene Codes). Assembled contigs were then verified, refined or corrected by mapping the 454 reads using GS Reference Mapper v2.6 (454 Life Sciences). Where needed, several rounds of manual assembly and trimming were performed in Sequencher with verification done using GS Reference Mapper to eliminate discrepancies or errors discovered during the prior reference mapping procedure.

### RT-PCR and Sanger Sequencing

Based on the assembled data obtained from NGS, primers for reverse transcription PCR (RT-PCR) and Sanger sequencing were designed (primer sequences available upon request). RT-PCR was performed with Superscript III reverse transcriptase (Life Technologies) and iProof DNA polymerase (Bio-Rad). The 3′ and 5′ termini of each genome RNA segment were amplified using both a 3′ and 5′ RACE approach based on ligation-anchored PCR, as previously described [36]–[38], with some sequences additionally confirmed using a commercially available 5′ RACE System (Life Technologies) according to the manufacturer's instructions.

### Sequence Alignment and Phylogenetic Analysis

The nucleotide sequences obtained for each genome segment, or the deduced amino acid sequences of each of the open reading frames, were aligned with the representative sequences of other known members of the genus *Orthobunyavirus* from GenBank (Table S1). Sequences were aligned using the MUSCLE algorithm and the evolutionary history for each tree construction was inferred using the neighbor-joining (NJ; [39]) and maximum likelihood methods (ML; [40]), as implemented in MEGA 5 [41]. For the NJ analyses, the evolutionary distances were computed using the Maximum Composite Likelihood method [42]. Statistical support for the tree topology obtained with all methods was evaluated based on bootstrap re-sampling [43] with values calculated based on 1,000 replicates.

### Comparison of Infection in Bat and Mosquito Cell Lines

RE05, Tb 1 Lu and C6/36 cells were grown for 80–90% confluence in 6 well plates, and the various virus strains indicated were used to infect these cells at an MOI of 0.1. The formation of CPE was monitored daily from 24–72 h and supernatants were harvested at 72 h post-infection for titration via plaque assay. Briefly, a 10-fold dilution series of supernatants were prepared in DMEM without FCS or supplements and 500 ul per well was applied to 12 well plates. Following incubation for 1 h at 37°C virus dilutions were removed and wells were overlaid with 0.9% agarose in 1× MEM containing 2% FCS, 2 mM L-glutamine, 50 U/ml penicillin and 50 µg/ml streptomycin. Once solidified plates were incubated at 37°C for 3 d (NDV(MP401), NDV (ERET147), MDCV and KKV) or 5 d (BWAV and PGAV) prior to fixation overnight in 10% formalin containing 0.1% crystal violet (Sigma-Aldrich).

### Nucleotide Sequence Accession Numbers

The genome sequences determined in this study were deposited in GenBank under the following accession numbers (S segment, M segment, and L segment): KJ867176, KJ867177 and KJ867178 (PGAV, strain SA Ar1); KJ867179, KJ867180 and KJ867181 (PGAV, strain 191B-07); KJ867182, KJ867183 and KJ867184 (BWAV, strain M459); KJ867185, KJ867186 and KJ867187 (BWAV, strain UgAr1888); KJ867188, KJ867189 and KJ867190 (NDV, strain MP401); KJ867191, KJ867192 and KJ867193 (NDV, strain UgAr1712); KJ867194, KJ867195 and KJ867196 (NDV, strain ERET147); KJ867197, KJ867198 and KJ867199 (NDV, strain YM176-66); KJ867200, KJ867201, and KJ867202 (MDCV, strain BeAn 276121); KJ867203, KJ867204 and KJ867205 (KKV, strain PSC-19).

## Results

### Phylogenetic Analysis of BWAV/PGAV and NDV Group Viruses

In order to better understand orthobunyavirus evolution as it relates to the relationships between and degree of diversity among BWAVs, PGAVs and NDVs, we have determined the complete genome sequences for all 3 viral RNA segments from multiple strains of each of these viruses. In addition, we have determined the complete sequences of a single strain of each of the “uncharacterized” bunyaviruses MDCV and KKV (listed in Table 1).

**Figure 2:**
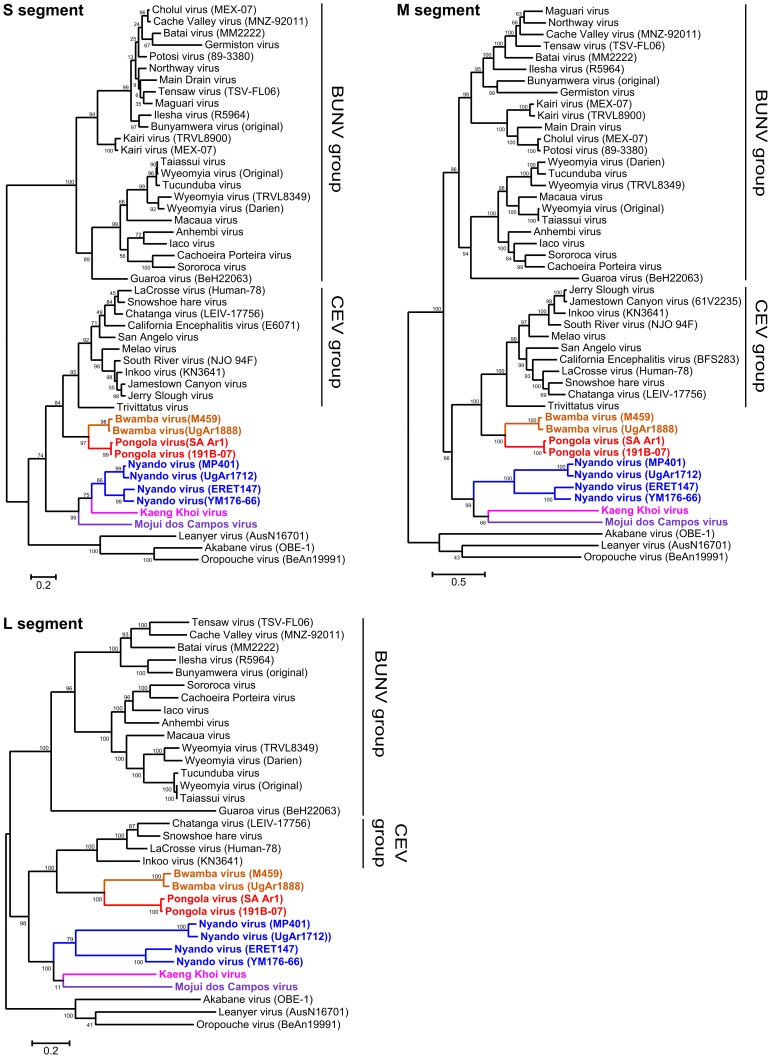
Phylogenetic relationships among BWAV/PGAV and NDV/MDCV/KKV group viruses. Maximum likelihood trees were constructed based on the nucleotide sequences of the S segment, M segment and L segment, as indicated. Bootstrap values based on 1,000 replicates are also indicated. Viruses lineages added based on sequences determined as a part of this study are indicated in color: Bwamba virus (orange), Pongola virus (red), Nyando virus (blue), Mojuí dos Campos virus (purple), Kaeng Khoi virus (pink).

Based on phylogenetic analysis of these viruses in relation to previously published data obtained from GenBank for other members of the genus *Orthobunyavirus* (Table S1), it is apparent that BWAV and PGAV form a distinct virus clade, separate from that formed by the NDV strains (Figure 2), consistent with their assignment to distinct serogroups. For BWAV and PGAV, all three segments form a lineage that diverged from a common ancestor shared with the California encephalitis virus (CEV) group, indicating that these two groups are closely related at the genetic level. The NDV clade is less closely related to any currently recognized orthobunyavirus group forming a very distinct genetic grouping branching ancestrally to both the CEV and BWAV/PGAV lineages. However, closer analysis of the NDV group also shows considerable variation between NDV strains, such that the NDV group actually forms two distinct clades: one composed of strains MP401 and UgAr1712, while the other is composed of strains ERET147 and YM176-66. The groupings and relative positions of these viruses were well-preserved regardless of whether a criterion-based method (ML; Figure 2) or a clustering method (NJ; Figure S1) was used for construction of the phylogenetic trees. Further, the same relationships are observed regardless of whether complete genome segment nucleotide sequence, coding region nucleotide sequence or amino acid sequence datasets are used for the analysis (data not shown).

### Genetic Identification of MDCV and KKV

Early serological data for MDCV had indicated a possible distant relationship to both CEV and/or bunyamwera group viruses. Further, a preliminary phylogenetic analysis of very short sequence data fragments available for KKV in GenBank (accession numbers JN010801 and AY843028–AY843038) indicated a possible, but weakly supported, evolutionary relationship to the NDV group (data not shown). On this basis we additionally undertook full-length sequencing of a single strain each of MDCV and KKV using *de novo* sequencing. Based on these complete genome data we found that both viruses clearly grouped together with the NDV viruses we had sequenced (Figure 2). However, despite NDV being the closest known relative of both of these viruses, at both the nucleotide and amino acid levels these viruses were considerably divergent from all NDV strains examined, demonstrating only 53–73% nucleotide identity and 39–70% amino acid identity for MDCV, and 53–75% nucleotide identity and 39–72% amino acid identity for KKV, clearly indicating that these should be considered as distinct virus species (Tables S2–S4).

### Analysis of Genome Structure and Homology

**Figure 3:**
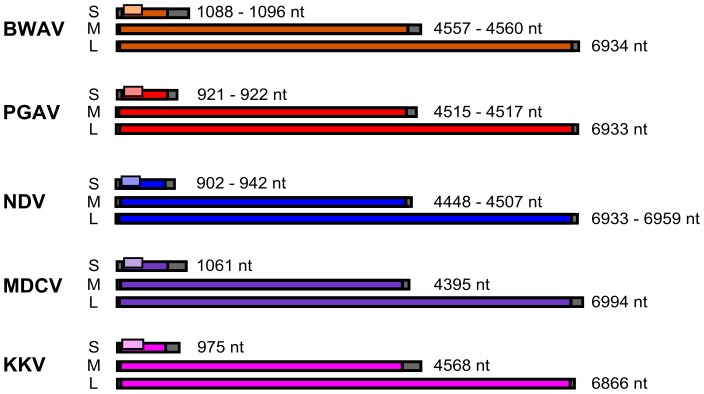
Comparison of virus genome structures. The genomes of the various virus groups sequenced in this study are shown to scale. The non-coding regions are shown in grey while the major open reading frames encoded by each segment [S segment: N (ORF), M segment: GPC (ORF) and L segment: LORF)] are shown in colored boxes. The NSs protein, which is produced from a downstream ATG in the S segment, is shown in a lighter shade of the corresponding color for each virus. The total genome length of each segment is indicated at right.

Analysis of the genome structures for all of these viruses indicates that they are consistent with what is known for previously analyzed orthobunyavirus genomes (Figure 3). The S segments for these viruses ranged from 902 [NDV (MP401)] to 1061 (MDCV) nucleotides and encoded both a nucleoprotein of 233 (NDV, MDCV, KKV) to 235 (PGAV) amino acids in length and an NS protein of 92 (BWAV, PGAV, NDV, MDCV) to 106 (KKV) amino acids derived from an alternate downstream ATG. The M segment and L segment were found to be between 4395 (MDCV) and 4568 (KKV) nucleotides, and 6866 (KKV) and 6994 (MDCV) nucleotides in length, respectively, and encoded only one long open reading frame (ORF) each, corresponding to the glycoprotein precursor (GPC) and the RNA-dependent RNA polymerase (L). The GPC protein of the various viruses were found to be between 1415 (MDCV) and 1447 (BWAV) nucleotides in length, while the L proteins were between 2249 (KKV) and 2268 (NDV) amino acids in length.

In order to apply rational criteria to the taxonomic assignments within these virus groups, we next analyzed the levels of sequence divergence between the different strains of BWAV, PGAV and NDV (Tables S2, S3, S4, S5, S6, S7). Based on these analyses we found 95–99% nucleotide identity among BWAV strains, while values for amino acid sequence conservation were between 98–99%. Among PGAV strains even higher levels of sequence identity were observed with 98%–99.6% sequence conservation at the nucleotide level and 99%–100% at the amino acid level. Despite the relationship between strains of either virus, sequence conservation between these two groups decreased to 67%–89% at the nucleotide level and 63%–86% at the amino acid level across all three segments. This clearly supports the classification of BWAV and PGAV as distinct virus species despite previous reports of strong serological cross-reactivity, which renders them nearly indistinguishable in some tests [5], [9], [16].

Among the NDVs, the situation observed was rather different. While the NDV clade is clearly highly divergent from all other recognized virus groups, it also demonstrated much more divergence between strains. Analysis showed that the MP401 and UgAr1712 strains exhibit 92%–99% identity at the nucleotide level and 96%–100% identity at the amino acid level, consistent with the levels of conservation observed between BWAV and PGAV strains. Similarly, the ERET147 and YM176-66 strains showed the expected high levels of sequence conservation, with 80%–98% identity at the nucleotide level and 90%–98% at the amino acid level. However, between these groups much more substantial differences in sequence were observed and identity levels dropped to 61–89% at the nucleotide level and 57%–86% at the amino acid level, respectively. These levels are then very similar to those seen when comparing BWAV and PGAV, members of two different virus species, and suggest that these two NDV clades should also be recognized as distinct orthobunyavirus species.

Given the paucity of full-length genome sequences available for bunyaviruses, including the orthobunyaviruses, little is known about the sequence and/or arrangement of their terminal untranslated regions (UTRs) (Figure S2). Based on a comparison of our full-length BWAV/PGAV and NDV/MDCV/KKV group sequences we noted that all sequences determined for these virus groups contained the well-conserved terminal sequences believed to be characteristic of all orthobunyavirus genome sequences (3′-AGTAGTGTAC…GCACACTACT-5′). In addition we found that the downstream 5 nt fit well to the sequences determined for Jamestown Canyon virus (JTCV), a member of the closely related California Encephalitis virus group. Further, where deviations from this prototype sequence were observed, compensatory mutations are found in the other UTR, which would then maintain base pairing at these positions. Such deviations from the established sequences of CEV group members were seen for the MDCV and KKV S segment UTRs and the BWAV and PGAV M segment UTRs (Figure S2). This observation supports previously proposed base pairing models, based on *in vitro* work, which have suggested direct interactions between the 3′ and 5′ UTR sequences that need to be maintained for functionality [44].

Beyond these well conserved terminal sequences we found that the UTR sequences exhibit a general lack of conservation in both sequence and length between different virus species. The 3′ UTRs varied in length from 24–85 nt (S segment: 38–85 nt, M segment: 24–49 nt, L segment: 27–45 nt), with the PGAV M segment 3′UTR being uncommonly long in comparison to other 3′ UTR sequences. Compared to the 3′ UTRs, the 5′ UTR sequences were generally much longer, ranging from 85–310 nt, and with sequences in excess of 200 nt determined for the BWAV, MDCV and KKV S segment 5′ UTRs and the KKV M segment 5′ UTR. Interestingly, despite the marked variability of these sequences between virus species, within a single species these sequences are in fact highly conserved. This can be clearly seen when examining the NDV(MP401) and NDV(UgAr1712) UTR sequences in comparison to those of NDV(ERET147) and NDV (YM176-66) (Figure S2). As such, both UTR sequence and length may provide useful additional criteria/markers for species delineation.

### Comparison of Infection in Bat and Mosquito Cell Lines

The genetic assignment of MDCV and KKV to the NDV clade was surprising, since neither of these viruses has been previously associated with transmission from mosquitos, which are the sole established vector for the transmission of BWAV, PGAV and NDV. In contrast, MDCV and KKV were both originally isolated from bats, which is rather unusual for orthobunyaviruses and has not been reported for BWAV, PGAV or NDV. However, it raises the possibility that these viruses might have a broader host/vector range than is currently appreciated.

**Figure 4:**
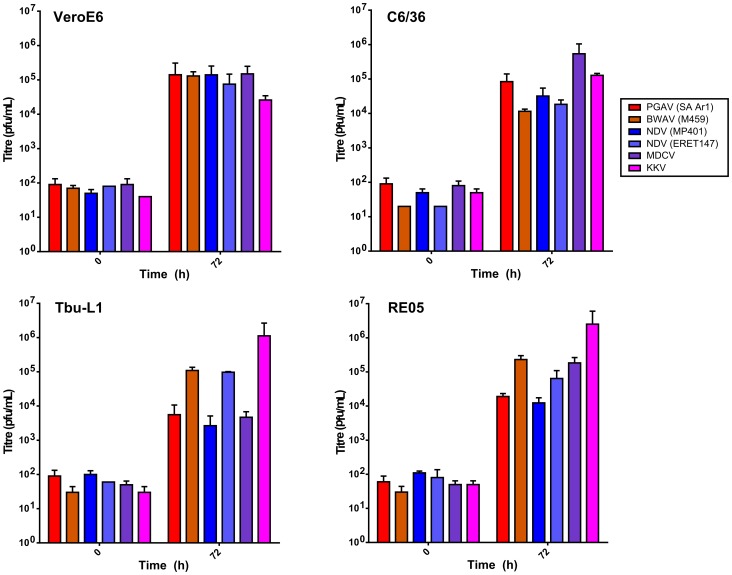
Comparison of virus growth in various cell lines. Cell lines derived from bats (i.e. Tb 1 Lu and RE05), mosquito (i.e. C6/36) or non-human primates (i.e. VeroE6) were infected with the indicated viruses at a multiplicity of infection of 0.1. Supernatants were harvested either immediately after infection (0 h) or after 72 h incubation and titres were determined using plaque assay.

In order to establish the feasibility of a role for these additional vector and host species in nature, we assessed the ability of representative viruses from the BWAV, PGAV, NDV, MDCV and KKV groups to productively infect cells from African (*Rousettus aegyptiacus*) and South American (*Tadarida brasiliensis*) bat species, as well as an *Aedes albopictus* mosquito cell line. Our data clearly indicate that all of these viruses have the ability to grow in both bat and mosquito cell types with titre increases in infected cells of between 1.5–4 logs between 0 and 72 hours post-infection (Figure 4). During the same time frame all of these viruses showed ∼3 logs of growth in VeroE6 cells, which are highly permissive for infection with a broad range of orthobunyavirus. There were no identifiable trends observed regarding which viruses (i.e. mosquito associated African orthobunyaviruses [BWAV, PGAV, NDV] or bat associated orthobunyaviruses from other regions [MDCV, KKV]) showed more efficient growth in any of these cell types. During infection, all of the viruses tested showed prominent cytopathic changes (CPE) in each of the mammalian cell types examined (i.e. VeroE6, RE05 and Tb 1 Lu cells; data not shown). In contrast, none of the viruses produced clear CPE in C6/36 cells. This lack of CPE in C6/36 cells occurred despite all viruses showing 3–4 logs of virus growth in these cells, comparable to what is seen, for instance, with VeroE6 cells where strong CPE formation is observed. Thus, it appears that this lack of CPE formation is a feature of infection in C6/36 cells rather than being influenced by the different viruses tested. Overall, based on these data it appears at least possible for KKV and MDCV to productively infect mosquito cells. Similarly infection of bat cells with BWAV, PGAV and NDV is also possible and leads to productive infection associated with cytopathological changes.

## Discussion

In this study we have determined the first full-length sequences for all three segments of multiple strains of BWAV and PGAV, as well as NDV, and for single strains of the related MDCV and KKV. As a result we have been able to definitively establish the relationships among these viruses, as well as their relationship to other orthobunyavirus groups. This work has not only clarified previously uncertain assessments about their relationships based on serology but has also led to the identification of two previously unclassified bunyaviruses, MDCV and KKV, as close relatives of NDV, and the identification of existing NDV strains as highly diverse, warranting classification into distinct virus species.

Our complete genome-based analysis of all three segments of BWAV and PGAV confirmed their placement within the orthobunyavirus genus, consistent with what has been previously reported based on S-segment analysis alone [45]. In particular, the position of these groups, branching immediately ancestral to the CEV group viruses for all three segments, explains previous reports of cross-reactivity of BWAV to members of the CEV group. Interestingly, despite their high degree of serological cross-reactivity in many assays, BWAV and PGAV display amino acid divergence values for all viral proteins that clearly support their classification as separate virus species. Indeed, the high degree of serological cross-reactivity between these viruses, including in neutralization assays [5], [9], [16], is surprising given that they exhibit only 64% amino acid identity in GPC. This suggests that there may be an unusually high degree of conservation among neutralizing epitopes in these viruses, in relation to the overall levels of sequence conservation, and/or that there may be a marked immunodominance of a few well conserved epitopes.

Unlike for BWAV and PGAV, the relative position identified by our analysis for NDV was not consistent with a previous report examining the S segment sequence of NDV (strain ArB16055) [21]. This previous study had suggested a close genetic relationship to Bunyamwera virus, an observation that was apparently at odds with serological evidence assigning NDV to a distinct serogroup [19]. In contrast, we clearly observed that all four of our Nyando virus isolates fell into a distinct clade ancestral to those formed by BWAV/PGAV and CEV and that this position was consistent for all three segments. We did not see any evidence for Nyando virus strains that were genetically related to bunyamwera virus on the S segment, suggesting that the identity of NDV (strain ArB16055) needs to be closely re-examined, as it may either have been misidentified or may be reassortant in nature. However, based on the currently available data, there does not appear to be any evidence for reassortment involving either the BWAV/PGAV or NDV groups.

In our study, very little genetic divergence was noted among different strains of BWAV and PGAV, despite substantial differences in the location and/or time at which they were collected, indicating that molecular detection methods based on information from one or a few virus strains may be adequate to detect all of the genetic diversity present for BWAV and PGAV. However, given the small number of isolates available for each of these virus species, we cannot exclude the possibility that other strains showing much more substantial sequence divergence also exists. In contrast to the situation for BWAV and PGAV, for NDV a large degree of genetic divergence was noted among the strains analyzed. Indeed, this genetic divergence was to such an extent that, based on a cut-off value of 10% amino acid divergence, a division of the existing NDV strains into two distinct virus species would be warranted. Given the prevailing naming conventions among bunyaviruses (i.e. naming based on the location of initial isolation) we would, therefore, propose that the prototype NDV (MP401), isolated from the Nyando river valley [18], and the closely related NDV (UgAr1712) continue to be referred to as “Nyando virus”, while NDV (ERET147), could be reclassified as “Manéra virus”, reflecting its original isolation from the Manéra forest in Ethiopia, along with the closely related NDV (YM176-66). Based on their close serological relatedness, it is also likely that other ERET and YM series viruses (e.g. ERET124, YM120-68 and YM259-68) isolated as part of the same studies [46], [47] will also fall into this genetic group. Our observation that NDV forms two highly distinct genetic groups supports early findings indicating that these viruses are serologically quite distinct [19]. Further, the closer genetic grouping of the ERET147 and YM176-66 strains is also supported by serological findings obtained during the initial isolation of YM176-66 [48].

The placement of MDCV within a larger NDV clade is also supported by early serological data which indicated reactivity by immunofluorescence assay (IFA) and complement fixation (CF), but not neutralization (NT), to both NDV and CEV group viruses [25]. Thus this is consistent with its genetic placement close to, but distinct from, both of these groups, as shown in this study. Initially, the existence of such a close relationship of these bat-associated bunyaviruses to what have been, until now, strictly mosquito-borne viruses was surprising. However, this finding appears to only contribute to the increasing number of bunyaviruses shown to be associated with bats. Interestingly, while for MDCV the virus has only been isolated from a single live bat with unreported health status [26], for KKV, infection in bats appears to be detrimental in a significant proportion of infected animals, as shown by frequent and consistent virus isolations from dead bats [28], [29]. Indeed, our *in vitro* data also indicate that bats should be more closely considered in future ecological and epidemiological investigations looking at BWAV, PGAV and NDV, as all of these viruses display at least a fundamental ability to infect cells derived from various bat species. Further, we cannot currently exclude that productive infection of bat cells may in fact be a feature of a wide range of other orthobunyavirus species as well, opening up the possibility that a broader host range than is currently appreciated might generally exist for orthobunyaviruses, and specifically that consideration of bats as a potential host for other orthobunyaviruses may also be warranted.

While for MDCV no arthropod vector has yet been established, for KKV, until now only bedbugs have been identified as a potential vector species [27]. On this basis, the classification of KKV in the NDV group was particularly surprising, since orthobunyavirus transmission appears to be almost exclusively mosquito-borne, with the exception of the Tete virus group, which is mainly transmitted by ticks, and a few specific instances of culicoid fly vectored viruses [1]. No other example of an orthobunyavirus associated with infection of and transmission via bedbugs has been reported. Interestingly, we also observed that both KKV and MDCV were able to infect mosquito cells *in vitro*, and while these data only show that infection of mosquitos is fundamentally possible at a cellular level, when taken together with the close genetic relationship of these viruses to a number of well-documented mosquito-borne viruses, this clearly indicates that the possibility of mosquito-borne transmission should be considered in future field studies. Such studies will be particularly important given that evidence exists supporting the relevance of KKV for human infection. In particular, high levels of seroprevalence among Guano miners working in caves known to have infected bats and/or bedbugs have been reported [28], [29], and anecdotal reports suggest that working in such caves is associated with a mild generalized febrile illness in new workers [27], [28].

The inclusion of MDCV and KKV in the NDV clade was also surprising given their geographical distribution, having been isolated exclusively from South America and Southeast Asia, respectively. The existence of such a highly genetically diverse clade, which spans three continents, suggests on the one hand that additional related but unrecognized virus species likely exist, and also that transmission between these non-contiguous geographical locations has most likely been facilitated by an unknown host species common to all of these virus groups. In particular, transmission of KKV and MDCV via migratory bird routes through various flyways would appear possible (i.e. via the American Flyways<>Black Sea and Mediterranean flyways<>Asia Flyways<>American Flyways), and indeed infection of bird species has been shown to be possible for several other orthobunyaviruses, including Turlock virus and Mermet virus [49], [50]. Alternatively, we must also consider that transmission between Africa and Asia may also have been facilitated by bats species endemic to these regions, some of which also populate broad geographical areas. In future the availability of comprehensive genome sequencing datasets, such as that determined in this study, will be important not only for molecular-based detection of virus infection (i.e. in infected mosquito samples, acutely infected humans, etc.) but will facilitate the development of recombinant antigen-based detection systems, which will be necessary for undertaking broader serological surveillance/screening efforts aimed at defining the geographic areas affected by these viruses, as well as estimating seroprevalence in animal species and/or larger segments of the human population to better define the public health impact of these viruses in the endemic areas. In addition, our study, which was restricted to only a small number of available isolates, also highlights the need for increased sample collection for these and other neglected tropical disease agents, and particularly the collection of human isolates, in order to develop a clearer picture of the actual extent of virus genetic diversity.

Overall the data contained in this study have not only led to the genetic identification of two previously uncharacterized viruses, but in doing so, has considerably expanded our knowledge of virus diversity along the BWAV/PGAV and NDV genetic lineages. On this basis we have also presented arguments for a more refined and evidence based approach to the taxonomic classification of the viruses in these groups, something that is increasingly appreciated as being sorely needed within the *Orthobunyavirus* genus. Further, our *in vitro* data, informed by the genetic relationships established as part of our sequencing efforts, have identified the possibility of infection with these viruses in an expanded range of host and vector species. The availability of complete genetic information for these viruses, as well as a better understanding of their genetic relationships, will be instrumental in assisting future surveillance efforts aimed at determining the distribution and public health impact of these viruses, as well as efforts in identifying the contributions of various potential host and vector species.

## Supporting Information

Figure S1 **Phylogenetic relationships among the BWAV/PGAV and NDV/MDCV/KKV group viruses as inferred using the Neighbor-Joining method.** Neighbor-Joining trees were constructed using the Tamura-3 parameter model on the nucleotide sequences of the S segment, M segment and L segment, as indicated. Bootstrap values based on 1,000 replicates are indicated. Viruses lineages added based on sequences determined as a part of this study are indicated in color: Bwamba virus (orange), Pongola virus (red), Nyando virus (blue), Mojuí dos Campos virus (purple), Kaeng Khoi virus (pink).
(TIF)

Figure S2 **Comparison of 3′ and 5′ untranslated region (UTR) sequences in BWAV/PGAV and NDV/MDCV/KKV group viruses.** The 3′ and 5′ UTR sequences are shown for the indicated viruses. For comparison, prototype members of the Bunyamwera virus group (Bunyamwera virus) and California Encephalitis virus group (Jamestown Canyon) are also shown. Previously identified highly conserved terminal sequences are highlighted in blue, while start/stop codons are indicated in green. Sequences that are predicted to form base-pairing interactions between the two UTRs (3′- 3 nt/8 nt/4 nt…4 nt/8 nt/3 nt- 5′) are delineated with black lines, and those nucleotides that differ from the CEV prototype sequence are indicated in red. Abbreviations: BUNV: Bunyamwera virus, BWAV: Bwamba virus, JTCV: Jamestown Canyon Virus, KKV: Kaeng Khoi virus, MDCV: Mojuí dos Campos virus, NDV: Nyando virus, PGAV: Pongola virus.
(TIF)

Table S1 **GenBank accession numbers for viruses used in the phylogenetic analyses.**
(DOCX)

Table S2 **Homology among N and NSs open reading frame sequences within the NDV clade.**
(DOCX)

Table S3 **Homology among GPC open reading frame sequences within the NDV clade.**
(DOCX)

Table S4 **Homology among L open reading frame sequences within the NDV clade.**
(DOCX)

Table S5 **Homology among N and NSs open reading frame sequences within the BWAV/PGAV clade.**
(DOCX)

Table S6 **Homology among GPC open reading frame sequences within the BWAV/PGAV clade.**
(DOCX)

Table S7 **Homology among L open reading frame sequences within the BWAV/PGAV clade.**
(DOCX)
