# Supplementary figures and images for: Molecular Characterization of Human Pathogenic Bunyaviruses of the Nyando and Bwamba/Pongola Virus Groups Leads to the Genetic Identification of Mojuí dos Campos and Kaeng Khoi Virus
Source: PLoS Negl Trop Dis. 2014 Sep 4;8(9):e3147. doi: 10.1371/journal.pntd.0003147 (PMC4154671; doi:10.1371/journal.pntd.0003147)

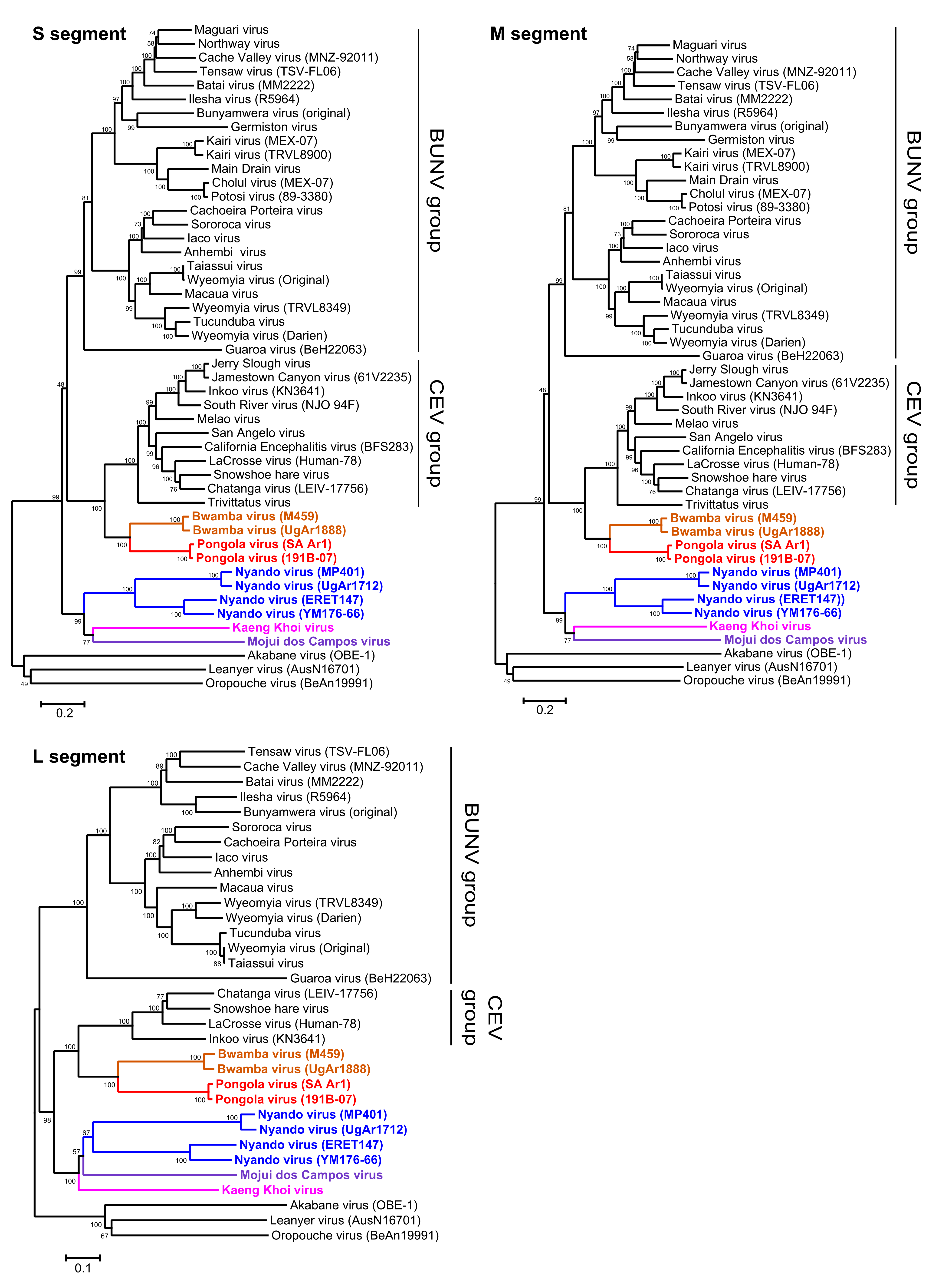

Supplement: Figure S1 — Phylogenetic relationships among the BWAV/PGAV and NDV/MDCV/KKV group viruses as inferred using the Neighbor-Joining method. Neighbor-Joining trees were constructed using the Tamura-3 parameter model on the nucleotide sequences of the S segment, M segment and L segment, as indicated. Bootstrap values based on 1,000 replicates are indicated. Viruses lineages added based on sequences determined as a part of this study are indicated in color: Bwamba virus (orange), Pongola virus (red), Nyando virus (blue), Mojuí dos Campos virus (purple), Kaeng Khoi virus (pink). (TIF) [file pntd.0003147.s001.tif]

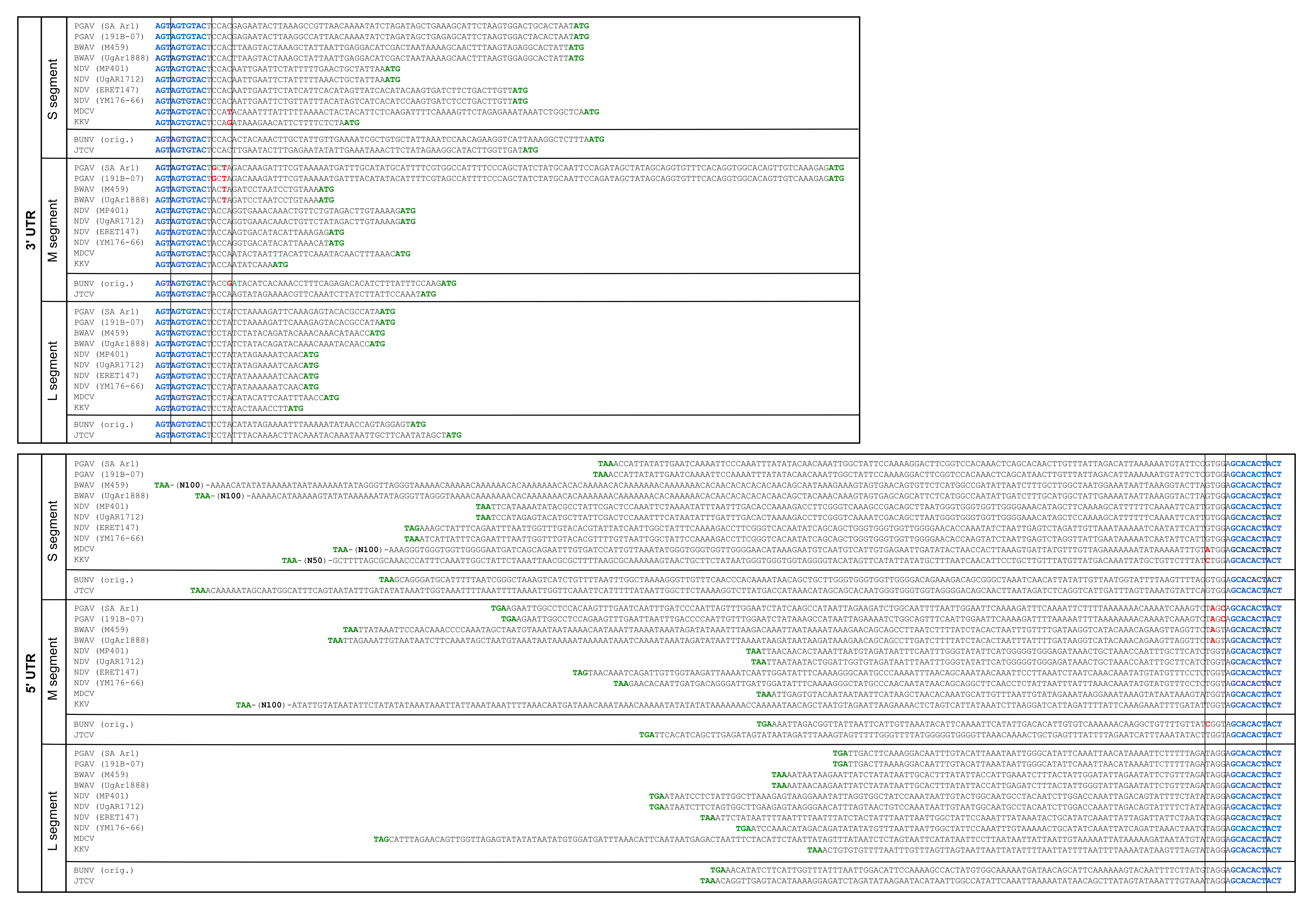

Supplement: Figure S2 — Comparison of 3′ and 5′ untranslated region (UTR) sequences in BWAV/PGAV and NDV/MDCV/KKV group viruses. The 3′ and 5′ UTR sequences are shown for the indicated viruses. For comparison, prototype members of the Bunyamwera virus group (Bunyamwera virus) and California Encephalitis virus group (Jamestown Canyon) are also shown. Previously identified highly conserved terminal sequences are highlighted in blue, while start/stop codons are indicated in green. Sequences that are predicted to form base-pairing interactions between the two UTRs (3′- 3 nt/8 nt/4 nt…4 nt/8 nt/3 nt- 5′) are delineated with black lines, and those nucleotides that differ from the CEV prototype sequence are indicated in red. Abbreviations: BUNV: Bunyamwera virus, BWAV: Bwamba virus, JTCV: Jamestown Canyon Virus, KKV: Kaeng Khoi virus, MDCV: Mojuí dos Campos virus, NDV: Nyando virus, PGAV: Pongola virus. (TIF) [file pntd.0003147.s002.tif]
