# Supplementary material for: Molecular Characterization of Human Pathogenic Bunyaviruses of the Nyando and Bwamba/Pongola Virus Groups Leads to the Genetic Identification of Mojuí dos Campos and Kaeng Khoi Virus
Source: PLoS Negl Trop Dis. 2014 Sep 4;8(9):e3147. doi: 10.1371/journal.pntd.0003147 (PMC4154671; doi:10.1371/journal.pntd.0003147)
Supplement: Table S1 — GenBank accession numbers for viruses used in the phylogenetic analyses. (DOCX) [file pntd.0003147.s003.docx]

**Table S1. GenBank accession numbers for viruses used in the phylogenetic**

| **Serogroup** | **Virus** | **Strain** | **GenBank Accession Numbers (S/M/L)** | | |
| --- | --- | --- | --- | --- | --- |
|  |  |  | **S segment** | **M segment** | **L segment** |
| **Bunyamwera** | Batai virus | MM2222 | JX846595 | JX846596 | JX846597 |
|  | Bunyamwera virus | Original | NC_001927 | NC_001926 | NC_001925 |
|  | Cache Valley virus | MNZ-92011 | KC436108 | KC436107 | KC436106 |
|  | Cholul virus | MEX-07 | EU879062 | JN808310 |  |
|  | Germiston virus | SAAr1050 | M19420 | M21951 |  |
|  | Ilesha virus | R5964 | AY729651 | KF234074 | KF234075 |
|  | Kairi virus | MEX-07 | EU879063 | GQ118699 |  |
|  | Kairi virus | TRVL8900 | X73467 | EU004186 |  |
|  | Maguari virus | BeAr7272 | D13783 | AY286443 |  |
|  | Main Drain virus | BFS5015 | X73469 | EU004187 |  |
|  | Northway virus | 0234 | X73470 | EU004188 |  |
|  | Potosi virus | 89-3380 | AY729652 | EU004189 |  |
|  | Tensaw virus | TSV-FL06 | FJ943507 | FJ943506 | FJ943509 |
| **California Encephalitis** | California Encephalitis virus | BFS283 | U12800 | AF123483 |  |
|  | Chatanga virus | LEIV-17756 | EU479697 | EU621834 |  |
|  | Inkoo virus | KN3641 | U47137 | U88059 | EU789573 |
|  | Jamestown Canyon virus | 61V2235 | U12796 | U88058 |  |
|  | Jerry Slough virus | BFS4474 | U12798 | AF123487 |  |
|  | LaCrosse virus | Human-78 | NC_004110 | NC_004109 | NC_004108 |
|  | Melao virus | TRVL9375 | U12802 | U88057 |  |
|  | San Angelo virus | VR723 | U47139 | AF123486 |  |
|  | Snowshoe hare virus | Original | EU294510 | EU262553 | EU203678 |
|  | South River virus | NJO 94F | U47141 | AF123488 |  |
|  | Trivittatus virus | Eklund | U12803 | AF123491 |  |
| **Simbu** | Leanyer virus | AusN16701 | HM627177 | HM627176 | HM627178 |
|  | Akabane virus | OBE-1 | NC_009896 | NC_009895 | NC_009894 |
|  | Oropouche virus | BeAn19991 | NC_005777 | NC_005775 | NC_005776 |

**analyses.**
