# Supplementary material for: Molecular Characterization of Human Pathogenic Bunyaviruses of the Nyando and Bwamba/Pongola Virus Groups Leads to the Genetic Identification of Mojuí dos Campos and Kaeng Khoi Virus
Source: PLoS Negl Trop Dis. 2014 Sep 4;8(9):e3147. doi: 10.1371/journal.pntd.0003147 (PMC4154671; doi:10.1371/journal.pntd.0003147)
Supplement: Table S2 — Homology among N and NSs open reading frame sequences within the NDV clade. (DOCX) [file pntd.0003147.s004.docx]

**Table S2. Homology among N and NSs open reading frame sequences within the NDV clade**

|  | **Nucleotide Identity (%)** | | | | | | |
| --- | --- | --- | --- | --- | --- | --- | --- |
| **Amino acid identity (%)** |  | **NDV**  **(MP401)** | **NDV**  **(UgAr 1712)** | **NDV**  **(ERET 147)** | **NDV**  **(YM 176-66)** | **MDCV**  **(BeAn276121)** | **KKV**  **(PSC-19)** |
|  | **NDV**  **(MP01)** |  | **96.0 / 98.6*** | **80.1 / 88.2** | **80.3 / 88.5** | **70.5 / 72.4** | **72.2 / 73.5** |
|  | **NDV**  **(UgAr 1712)** | **100.0 / 95.7** |  | **78.5 / 86.6** | **79.8 / 87.0** | **70.8 / 72.8** | **72.4 / 72.1** |
|  | **NDV**  **(ERET 147)** | **85.5 / 81.7** | **85.5 / 77.2** |  | **88.9 / 97.8** | **67.4 / 71.3** | **72.6 / 75.3** |
|  | **NDV**  **(YM 176-66)** | **84.6 / 82.8** | **84.6 / 78.3** | **98.3 / 96.8** |  | **68.7 / 71.3** | **72.8 / 76.0** |
|  | **MDCV**  **(BeAn276121)** | **69.7 / 55.9** | **69.7 / 57.6** | **68.4 / 51.6** | **68.4 / 51.6** |  | **67.5 / 68.8** |
|  | **KKV**  **(PSC-19)** | **72.2 / 61.3** | **72.2 / 57.6** | **71.4 / 63.4** | **71.4 / 64.5** | **67.1 / 49.5** |  |

*Values are shown for N (ORF) / NSs (ORF)
