# Supplementary material for: Molecular Characterization of Human Pathogenic Bunyaviruses of the Nyando and Bwamba/Pongola Virus Groups Leads to the Genetic Identification of Mojuí dos Campos and Kaeng Khoi Virus
Source: PLoS Negl Trop Dis. 2014 Sep 4;8(9):e3147. doi: 10.1371/journal.pntd.0003147 (PMC4154671; doi:10.1371/journal.pntd.0003147)
Supplement: Table S3 — Homology among GPC open reading frame sequences within the NDV clade. (DOCX) [file pntd.0003147.s005.docx]

**Table S3. Homology among GPC open reading frame sequences within the NDV clade**

|  | **Nucleotide Identity (%)** | | | | | | |
| --- | --- | --- | --- | --- | --- | --- | --- |
| **Amino acid identity (%)** |  | **NDV**  **(MP401)** | **NDV**  **(UgAr 1712)** | **NDV**  **(ERET 147)** | **NDV**  **(YM 176-66)** | **MDCV**  **(BeAn276121)** | **KKV**  **(PSC-19)** |
|  | **NDV**  **(MP01)** |  | **92.0** | **61.6** | **62.1** | **52.8** | **53.6** |
|  | **NDV**  **(UgAr 1712)** | **95.7** |  | **61.2** | **61.9** | **52.5** | **53.1** |
|  | **NDV**  **(ERET 147)** | **57.3** | **56.9** |  | **79.7** | **53.0** | **54.3** |
|  | **NDV**  **(YM 176-66)** | **57.0** | **56.6** | **90.2** |  | **53.2** | **54.8** |
|  | **MDCV**  **(BeAn276121)** | **39.9** | **39.4** | **40.9** | **40.7** |  | **52.6** |
|  | **KKV**  **(PSC-19)** | **39.5** | **39.0** | **40.3** | **40.3** | **38.2** |  |
