# Supplementary material for: Molecular Characterization of Human Pathogenic Bunyaviruses of the Nyando and Bwamba/Pongola Virus Groups Leads to the Genetic Identification of Mojuí dos Campos and Kaeng Khoi Virus
Source: PLoS Negl Trop Dis. 2014 Sep 4;8(9):e3147. doi: 10.1371/journal.pntd.0003147 (PMC4154671; doi:10.1371/journal.pntd.0003147)
Supplement: Table S4 — Homology among L open reading frame sequences within the NDV clade. (DOCX) [file pntd.0003147.s006.docx]

**Table S4. Homology among L open reading frame sequences within the NDV clade**

|  | **Nucleotide Identity (%)** | | | | | | |
| --- | --- | --- | --- | --- | --- | --- | --- |
| **Amino acid identity (%)** |  | **NDV**  **(MP401)** | **NDV**  **(UgAr 1712)** | **NDV**  **(ERET 147)** | **NDV**  **(YM 176-66)** | **MDCV**  **(BeAn276121)** | **KKV**  **(PSC-19)** |
|  | **NDV**  **(MP01)** |  | **92.6** | **62.8** | **62.8** | **61.2** | **61.5** |
|  | **NDV**  **(UgAr 1712)** | **96.7** |  | **63.0** | **62.6** | **61.0** | **61.2** |
|  | **NDV**  **(ERET 147)** | **61.9** | **62.3** |  | **81.7** | **63.9** | **65.2** |
|  | **NDV**  **(YM 176-66)** | **61.6** | **62.0** | **92.8** |  | **63.7** | **65.2** |
|  | **MDCV**  **(BeAn276121)** | **57.8** | **57.8** | **59.7** | **62.0** |  | **63.0** |
|  | **KKV**  **(PSC-19)** | **59.7** | **59.6** | **62.0** | **62.1** | **60.0** |  |
