# Supplementary material for: Molecular Characterization of Human Pathogenic Bunyaviruses of the Nyando and Bwamba/Pongola Virus Groups Leads to the Genetic Identification of Mojuí dos Campos and Kaeng Khoi Virus
Source: PLoS Negl Trop Dis. 2014 Sep 4;8(9):e3147. doi: 10.1371/journal.pntd.0003147 (PMC4154671; doi:10.1371/journal.pntd.0003147)
Supplement: Table S5 — Homology among N and NSs open reading frame sequences within the BWAV/PGAV clade. (DOCX) [file pntd.0003147.s007.docx]

**Table S5. Homology among N and NSs open reading frame sequences within the BWAV/PGAV clade**

|  | **Nucleotide Identity (%)** | | | | |
| --- | --- | --- | --- | --- | --- |
| **Amino acid identity (%)** |  | **BWAV**  **(M459)** | **BWAV**  **(UgAr 1888)** | **PGAV**  **(SA AR 1)** | **PGAV**  **(191B-07)** |
|  | **BWAV**  **(M459)** |  | **97.7 / 99.3** | **80.1 / 87.8** | **80.7 / 88.2** |
|  | **BWAV**  **(UgAr 1888)** | **99.1 / 98.9** |  | **80.3 / 88.5** | **80.9 / 88.9** |
|  | **PGAV**  **(SA AR 1)** | **86.0 / 73.1** | **86.4 / 73.1** |  | **99.4 / 99.6** |
|  | **PGAV**  **(191B-07)** | **86.0 / 74.2** | **86.4 / 74.2** | **100 / 98.9** |  |

*Values shown for N (ORF) / NSs (ORF)
