# Supplementary material for: Molecular Characterization of Human Pathogenic Bunyaviruses of the Nyando and Bwamba/Pongola Virus Groups Leads to the Genetic Identification of Mojuí dos Campos and Kaeng Khoi Virus
Source: PLoS Negl Trop Dis. 2014 Sep 4;8(9):e3147. doi: 10.1371/journal.pntd.0003147 (PMC4154671; doi:10.1371/journal.pntd.0003147)
Supplement: Table S6 — Homology among GPC open reading frame sequences within the BWAV/PGAV clade. (DOCX) [file pntd.0003147.s008.docx]

**Table S6. Homology among GPC open reading frame sequences within the BWAV/PGAV clade**

|  | **Nucleotide Identity (%)** | | | | |
| --- | --- | --- | --- | --- | --- |
| **Amino acid identity (%)** |  | **BWAV**  **(M459)** | **BWAV**  **(UgAr 1888)** | **PGAV**  **(SA AR 1)** | **PGAV**  **(191B-07)** |
|  | **BWAV**  **(M459)** |  | **95.2** | **67.1** | **67.1** |
|  | **BWAV**  **(UgAr 1888)** | **97.6** |  | **67.4** | **67.2** |
|  | **PGAV**  **(SA AR 1)** | **64.0** | **64.3** |  | **97.5** |
|  | **PGAV**  **(191B-07)** | **64.2** | **64.4** | **99.1** |  |
