# Supplementary material for: Molecular Characterization of Human Pathogenic Bunyaviruses of the Nyando and Bwamba/Pongola Virus Groups Leads to the Genetic Identification of Mojuí dos Campos and Kaeng Khoi Virus
Source: PLoS Negl Trop Dis. 2014 Sep 4;8(9):e3147. doi: 10.1371/journal.pntd.0003147 (PMC4154671; doi:10.1371/journal.pntd.0003147)
Supplement: Table S7 — Homology among L open reading frame sequences within the BWAV/PGAV clade. (DOCX) [file pntd.0003147.s009.docx]

**Table S7. Homology among L open reading frame sequences within the BWAV/PGAV clade**

|  | **Nucleotide Identity (%)** | | | | |
| --- | --- | --- | --- | --- | --- |
| **Amino acid identity (%)** |  | **BWAV**  **(M459)** | **BWAV**  **(UgAr 1888)** | **PGAV**  **(SA AR 1)** | **PGAV**  **(191B-07)** |
|  | **BWAV**  **(M459)** |  | **94.6** | **70.8** | **70.8** |
|  | **BWAV**  **(UgAr 1888)** | **97.6** |  | **70.9** | **71.1** |
|  | **PGAV**  **(SA AR 1)** | **75.1** | **75.3** |  | **98.2** |
|  | **PGAV**  **(191B-07)** | **75.1** | **75.2** | **99.5** |  |
